# Rational Modification of a Metallic Substrate for CVD Growth of Carbon Nanotubes

**DOI:** 10.1038/s41598-018-22467-7

**Published:** 2018-03-12

**Authors:** Xu Li, Montgomery Baker-Fales, Haider Almkhelfe, Nolan R. Gaede, Tyler S. Harris, Placidus B. Amama

**Affiliations:** Department of Chemical Engineering, Kansas State University, Manhattan, KS 66506 USA

## Abstract

Growth of high quality, dense carbon nanotube (CNT) arrays via catalytic chemical vapor deposition (CCVD) has been largely limited to catalysts supported on amorphous alumina or silica. To overcome the challenge of conducting CNT growth from catalysts supported on conductive substrates, we explored a two-step surface modification that involves ion beam bombardment to create surface porosity and deposition of a thin Al_x_O_y_ barrier layer to make the surface basic. To test the efficacy of our approach on a non-oxide support, we focus on modification of 316 stainless steel (SS), a well-known inactive substrate for CNT growth. Our study reveals that ion beam bombardment of SS has the ability to reduce film thickness of the Al_x_O_y_ barrier layer required to grow CNTs from Fe catalysts to $\sim$ 5 nm, which is within the threshold for the substrate to remain conductive. Additionally, catalysts supported on ion beam-damaged SS with the same Al_x_O_y_ thickness show improved particle formation, catalyst stability, and CNT growth efficiency, as well as producing CNTs with higher quality and density. Under optimal reaction conditions, this modification approach can lead to CNT growth on other nontraditional substrates and potentially benefit applications that require CNTs be grown on a conductive substrate.

## Introduction

To harness the unique properties of carbon nanotube (CNT) arrays for applications in energy storage and thermal management, it is necessary for them to be supported on metallic substrates. Hitherto, efficient growth of high quality, dense CNT arrays via catalytic chemical vapor deposition (CCVD) occurs mainly from a catalyst supported on an insulator such as amorphous alumina (Al_x_O_y_) or silica, which is usually unsuitable in applications that require conductive substrates such as energy storage^1,2^, thermal interface materials^3,4^, and sensing devices^5,6^. On the other hand, extending the high CNT growth efficiency observed on alumina to non-alumina supports such as metals has remained a challenge for a number of reasons. First, high surface energies of metallic substrates hamper formation of catalyst nanoparticles during the annealing process^7–9^. Second, metallic substrates are unable to stabilize the catalyst and prevent high rates of intermetallic diffusion and catalyst poisoning that typically occurs during CNT growth^10–12^. Therefore, significant interest exists in development of rational modification approaches that transform nontraditional “inactive” substrates to “active” substrates with physicochemical properties that support high CNT nucleation and growth.

CNT growth on metallic substrates such as Al^13^, Cu^14^, Inconel^15^, and stainless steel (SS)^16^ has been achieved through implementation of different strategies. A commonly used strategy involves depositing a barrier layer, such as Ti^14^ or Al_2_O_3_^15–17^, that is sandwiched between a layer of catalyst (Ni^13,14^ or Fe^15,16^) at the top and metallic substrate at the bottom. The barrier layer prevents direct interaction of the catalyst with the underlying metal to prevent the problems delineated above. For metallic substrates that contain an active catalyst for CNT growth such as SS and Inconel, it is possible to grow CNTs on them directly (without deposition of a catalyst) after conducting specific surface modification or pretreatment. Pretreatment methods investigated include acid-etching using HCl^16,18^ or H_2_SO_4_^19^, plasma treatment^20,21^, air annealing^22–24^, Ar ion bombardment,^25^ and magnetron sputtering^26^. All these methods favor formation of catalyst particles on the substrate that serve as seeds for CNT growth. Based on studies involving CNT growth on different types of SS (316 SS and 304 SS), catalytic activity of pretreated SS during CNT growth shows high sensitivity to the composition of SS. The type of SS used in our study is 316 SS, which is alloyed with 2–3% Mo to enhance its corrosion resistance and thermal stability^24^. Presence of Mo in 316 SS makes it a relatively inactive substrate for CNT growth^27^. Conversely, 304 SS contains no Mo and is active for CNT growth; in fact, a number of prior studies^16,19,22^ involving direct CNT growth on SS utilize 304 SS as growth substrate.

**Figure 1:**
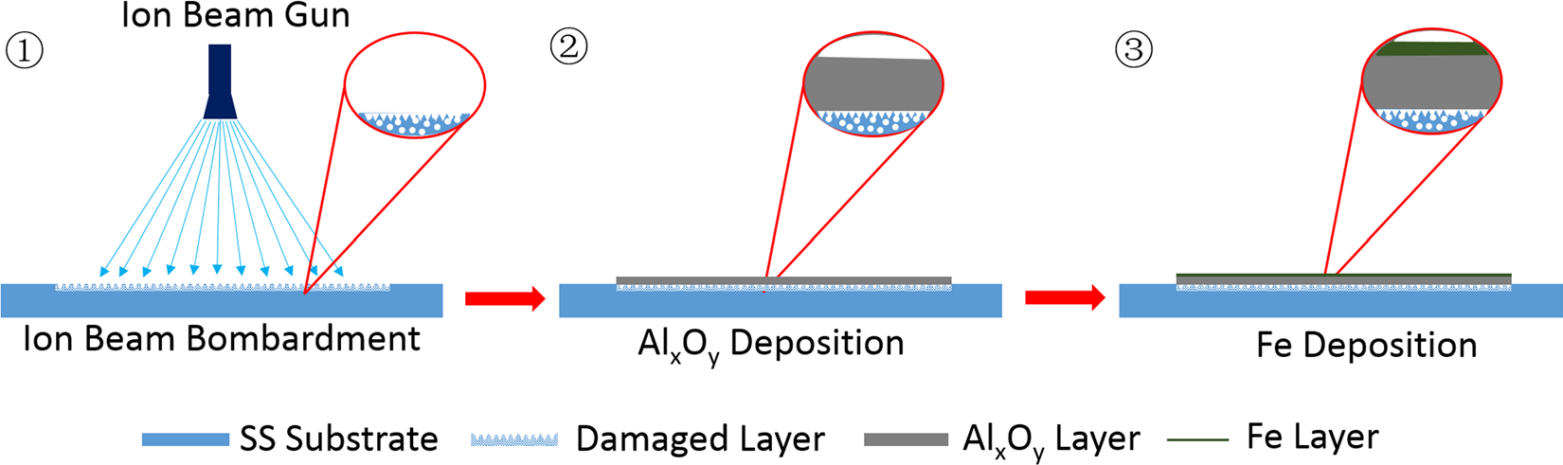
A schematic illustration of ion beam bombardment of SS (Step 1), Al_x_O_y_ deposition on the damaged surface (Step 2) followed by Fe deposition (Step 3).

In this study, we focus on modification of 316 SS, a well-known inactive substrate for CNT growth, via a two-step process involving ion beam bombardment and deposition of a thin Al_x_O_y_ layer (Fig. 1). The modification approach implemented in this study results in growth of CNT arrays on 316 SS. The approach is inspired by previous investigations^28–32^ on the role of Al_x_O_y_ as a catalyst support in CNT carpet growth, whereby the high growth efficiency observed for a supported Fe catalyst was attributed to the combined effect of surface porosity and Lewis basicity of Al_x_O_y_. In particular, our results show that ion beam bombardment of 316 SS decreases the film thickness of Al_x_O_y_ required for CNT growth to 5 nm Al_x_O_y_, a thickness that does not impede electronic transport across the barrier^17,33^. In elucidating the role of ion beam bombardment in CNT growth, we have focused on probing the evolution of the catalyst during CCVD, given that prior studies^34–37^ have illuminated the effect of ion beam bombardment on substrates including SS.

## Results

### Modification of SS

Surface porosity and basicity on 316 SS were created via a two-step process (Steps 1 and 2 in Fig. 1) prior to catalyst deposition (Step 3). In Step 1, the 316 SS substrates used as catalyst supports were modified by ion beam bombardment in an ion beam sputter deposition and etching system, while in Step 2, the damaged and pristine SS substrates were deposited with different thicknesses of Al_x_O_y_ films (0, 5, 10, and 20 nm). A thin Fe catalyst film with a nominal thickness of 1 nm was then deposited on each SS-supported Al_x_O_y_ layer. Note that Al_x_O_y_ and Fe films were sequentially deposited on two sets of SS samples: (1) Fe deposited on ion beam-damaged SS substrate with different thicknesses of Al_x_O_y_: 0 nm Al_x_O_y_ (Damaged-0), 5 nm Al_x_O_y_ (Damaged-5), 10 nm Al_x_O_y_ (Damaged-10), and 20 nm Al_x_O_y_ (Damaged-20). (2) Fe deposited on pristine SS substrate with different thicknesses of Al_x_O_y_: 0 nm Al_x_O_y_ (Pristine-0), 5 nm Al_x_O_y_ (Pristine-5), 10 nm Al_x_O_y_ (Pristine-10), and 20 nm Al_x_O_y_ (Pristine-20). The samples are hereinafter referred to by the condition of the SS surface (damaged or pristine) followed by thickness of the Al_x_O_y_ barrier layer (in nanometers) as stated above in parentheses.

### Effects of ion beam bombardment and Al_x_O_y_ thickness on CNT growth

**Figure 2:**
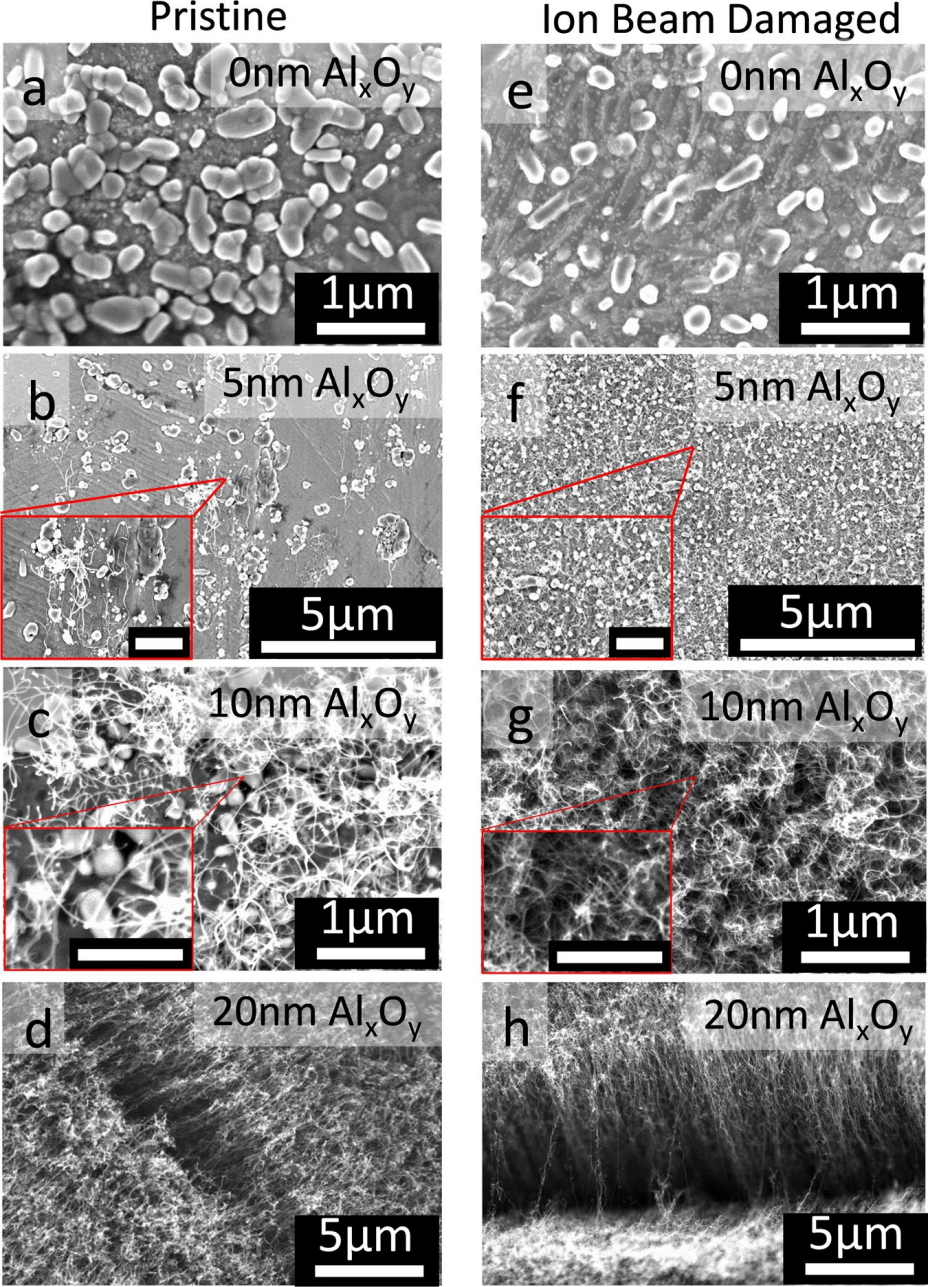
SEM characterization of SS surface after CCVD growth. Images of products formed on pristine and ion beam-damaged SS-supported Fe catalyst with different thicknesses of Al_x_O_y_ after CNT growth process: (**a**) Pristine-0, (**b**) Pristine-5, (**c**) Pristine-10, (**d**) Pristine-20, (**e**) Damaged-0, (**f**) Damaged-5, (**g**) Damaged-10, and (**h**) Damaged-20. Scale bars of inserts in panels b and f are 1 µm while those of panels c and g are 500 nm.

Figure 2 shows SEM images of products formed after CCVD on pristine and ion beam-damaged substrates with different Al_x_O_y_ barrier layer thicknesses sandwiched between Fe catalysts and SS. Panel a in Fig. 2 shows large Fe particles with no CNTs on Pristine-0. Panel b shows poor and non-uniform CNT growth on Pristine-5, and the Fe catalyst particles appear to be covered with amorphous carbon as verified by Raman spectroscopy. For pristine SS samples, uniform and efficient CNT growth from Fe catalysts becomes apparent on Pristine-10 and denser on Pristine-20, as shown in panels c and d of Fig. 2, respectively. In contrast, panel f shows a catalyst on damaged substrates start to grow CNTs with a barrier layer thickness of only 5 nm (Damaged-5), and become denser with increasing barrier layer thickness (Damaged-10 and Damaged-20). Although CNT density and growth uniformity was higher on Damaged-5 than Pristine-5, the density was still lower than what is required to create CNT carpets. Al_x_O_y_ layer thickness of 20 nm was required for CNT carpet growth on damaged and pristine SS, albeit with higher CNT density on the former. A related work by Hiraoka *et al*.^17^ demonstrated growth of single- and double-wall CNTs from catalyst supported on nickel alloy, 304 SS, and 310 SS substrates, albeit with a thicker alumina barrier layer (30 nm), further illustrating the difficulty in achieving CNT growth on metallic substrates with a thin barrier layer. SEM images of pristine and damaged SS demonstrate that surface modification via ion beam bombardment can in fact decrease the barrier layer thickness required for CNT growth, which may explain the successful growth of unaligned (5–10 nm-thick Al_x_O_y_) and vertically aligned CNTs (20 nm-thick Al_x_O_y_) on thinner alumina layers in our study. As shown by Zhong *et al*.^33^, a thin barrier thickness of $\le$ 5 nm is within the threshold for the substrate to remain conductive. Therefore, with optimization of reaction conditions for each catalyst, this new approach can potentially benefit applications that require CNT arrays supported on a conductive substrate.

**Figure 3:**
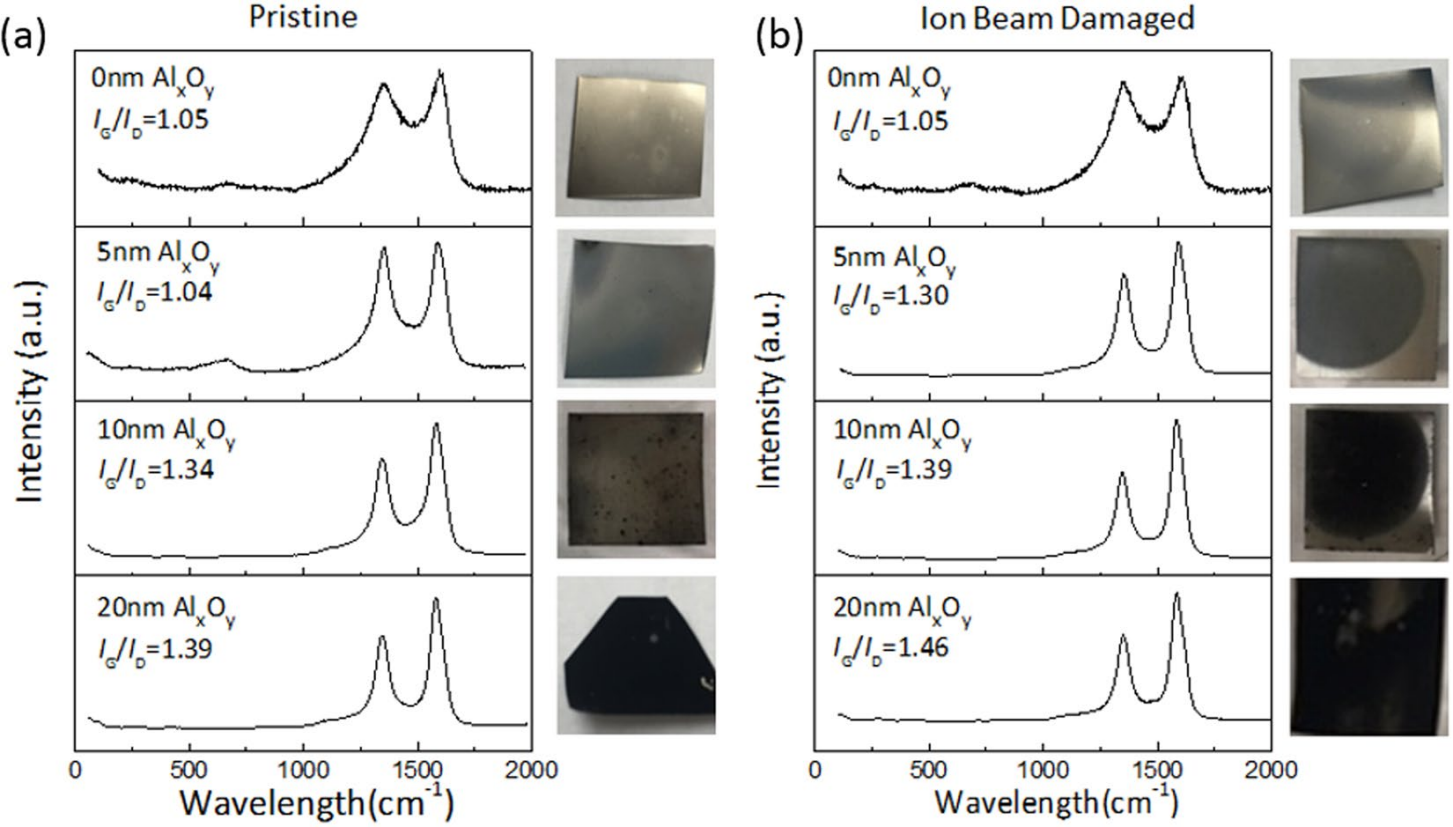
Characterization of products formed on pristine and ion beam-damaged SS. Raman spectra of products formed on pristine SS-supported Fe catalysts with different thicknesses of Al_x_O_y_ barrier layer (**a**) and ion beam-damaged SS-supported Fe catalysts with different thicknesses of Al_x_O_y_ barrier layer (**b**).

The Raman spectra of products formed on the surface of pristine and ion beam-damaged substrates with different Al_x_O_y_ barrier layer thicknesses after a CCVD process are shown in Fig. 3. A photograph of the sample surface is placed beside each spectrum to show coverage of the growth product on SS. The Raman spectra show characteristic modes of CNTs: tangential stretch mode (G-band) at ~1593 cm^−1^ that represents the highly oriented lattice structure of graphitic carbon, and disorder-induced mode (D-band) around 1345 cm^−1^ that is indicative of the presence of defects or amorphous carbon. The ratio of G-band to D-band intensities (*I*_*G*_*/I*_*D*_) has been used as an index to evaluate the quality of the grown CNTs, and the value for each spectrum is provided in Fig. 3. Additionally, a shoulder on the right side of the G-band (~1600 cm^−1^) is referred to as the D’ line and is also indicative of disorders in the graphitic crystal structure^38,39^. Raman spectra of Pristine-0, Pristine-5, and Damaged-0 samples have an additional peak at 660 cm^−1^, which comes from the SS substrate. Unlike Pristine-0, Pristine-5, and Damaged-0, absence of the D′ peak and the peak at 660 cm^−1^ (attributed to SS) in the spectrum of Damaged-5 supports evidence from SEM data (Fig. 2) that shows improved CNT growth on Damaged-5, due to the combined effect of ion beam bombardment and presence of a thin Al_x_O_y_ layer.

Pictures shown on the side of the Raman spectra reveal that carbon deposition during CCVD was limited to the circular area that had been ion beam damaged even though the catalyst was deposited across the entire substrate; this result emphasizes the critical role played by ion beam bombardment in combination with a basic surface. Ion beam bombardment can induce changes in surface and diffusion properties of the substrate as well as composition due to vacancies that may be created. From Fig. 2, coverage of CNTs on pristine and damaged SS increases with barrier-layer thickness, which based on previous studies correlates with increased surface porosity and the basic environment provided by the Al_x_O_y_ barrier layer^28–30,32^. Also, from the Raman data in Fig. 3, *I*_*G*_*/I*_*D*_ for CNTs grown on pristine and damaged SS increase with Al_x_O_y_ barrier layer thickness, indicating the quality of CNTs improves with increasing Al_x_O_y_ thickness. Substrate basicity has been shown to enhance nanoparticle formation and stabilization during annealing and growth steps^29,40–42^. Note that for the same Al_x_O_y_ thickness, CNTs grown on damaged substrates have higher coverage and *I*_*G*_*/I*_*D*_, which demonstrates that ion beam bombardment enhances CNT growth efficiency from Fe catalysts. We observed similar improvement in growth efficiency on c-cut sapphire substrates after ion beam bombardment, which was attributed to changes in Lewis basicity, porosity, and damage depth of substrates^28^. Note that unlike c-cut sapphire, in the present study, ion beam damage alone does not transform SS to an “active” catalyst support for CNT growth, as evidenced by the lack of growth on Damaged-0. In fact, Raman spectra of Damaged-0, Pristine-0, and Pristine-5 after CNT growth are characterized by G- and D-bands with relatively low signal-to-noise ratio and a signal ~660 cm^−1^ from the background; we attribute this observation to the low amount of carbon formed, causing most of the signal to emanate from the substrate.

### CNT growth rate and density

**Figure 4:**
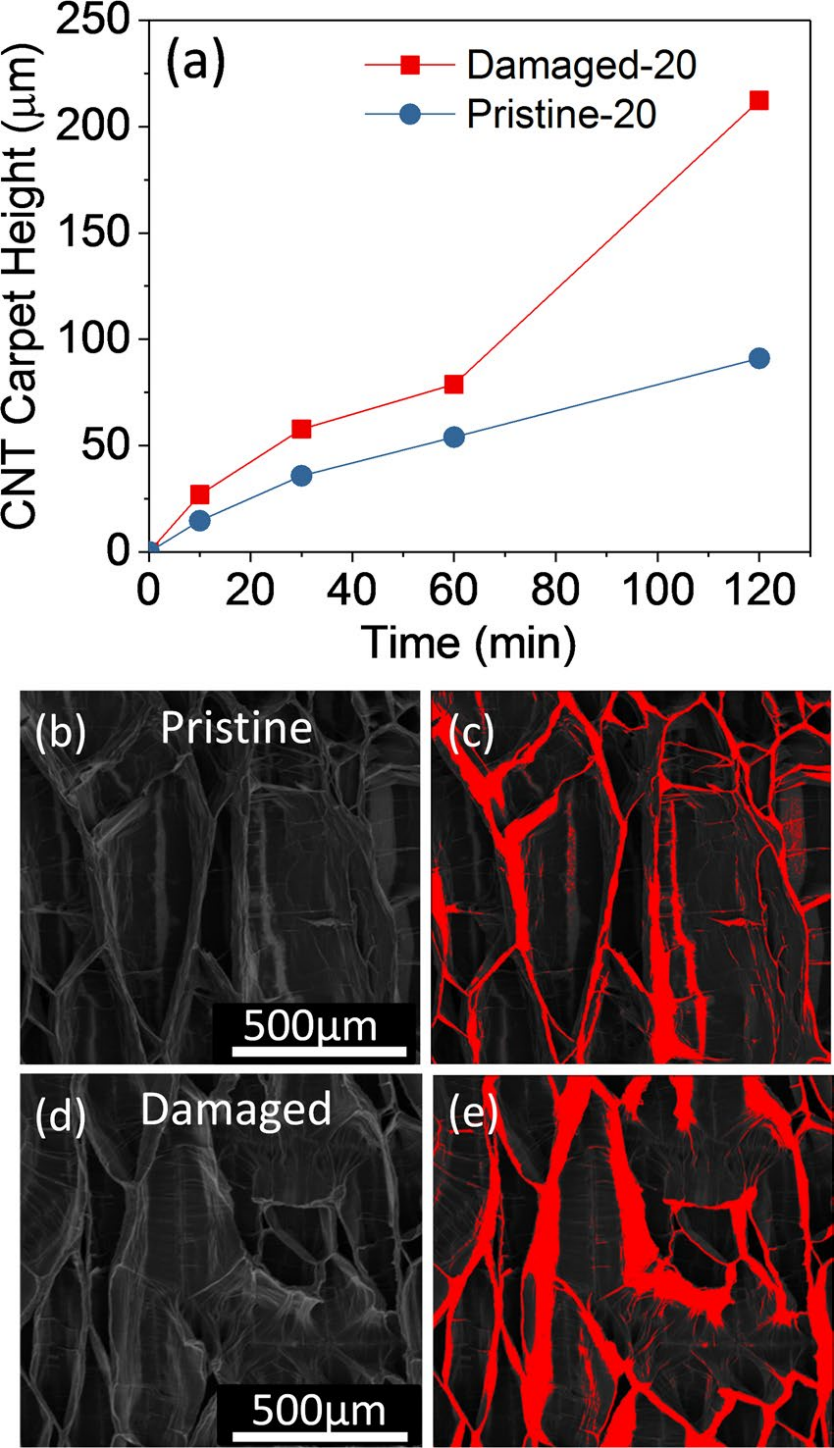
Growth properties of CNT carpets on pristine and ion beam-damaged SS. Plots of CNT carpet height as a function of growth time for Pristine-20 and Damaged-20 (**a**). SEM image of densified CNT carpets grown for 120 min from Fe catalyst supported on Pristine-20 (**b**) and its corresponding false-color image showing the densified region (**c**). SEM image of densified CNT carpets grown for 120 min from Fe catalyst supported on Damaged-20 (**d**) and its corresponding false-color image showing the densified region (**e**).

To investigate the effect of ion beam bombardment of SS on CNT growth efficiency, height of CNT carpets grown on Pristine-20 and Damaged-20 for various growth times, ranging from 10 to 120 min, were investigated. The results, presented in Fig. 4, reveal that CNT carpets grown on Damaged-20 are taller than carpets grown on Pristine-20 especially at longer growth time (> 60 min) when catalysts become prone to deactivation. Growth rates of CNT carpets from catalysts supported on Pristine-20 and Damaged-20 catalysts are 1.08 µm/min and 1.92 µm/min, respectively. Modification of SS via ion beam bombardment enhances CNT growth rate by almost a factor of two. It is noteworthy that the positive impact of ion beam bombardment is still apparent for a barrier-layer thickness of 20 nm, suggesting the influential role it plays in CNT growth.

**Figure 5:**
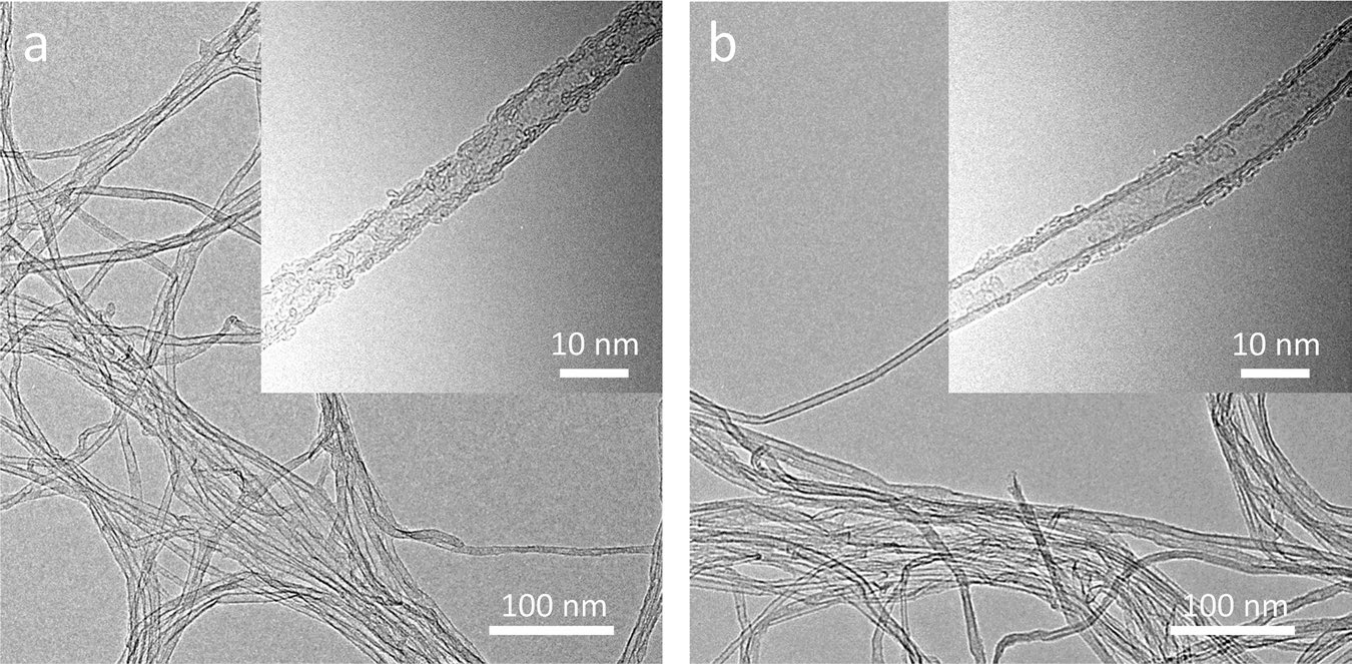
TEM images of CNTs after 120 min of growth. (**a**) CNT arrays grown from Fe catalyst supported on Pristine-20; the insert shows the defective wall of a CNT. (**b**) CNT arrays grown from Fe catalyst supported on Damaged-20; the insert shows a wall structure that has less defects.

To determine density of as-grown CNT carpets on Pristine-20 and Damaged-20 catalysts, we employed a solvent-induced densification method. The process involves soaking CNT carpets in ethanol, followed by drying in air. Thereafter, the CNT carpets were densified via capillary forces^43^. SEM images of the top view of CNTs grown on Pristine-20 and Damaged-20 catalyst are compared in Fig. 4b–d, respectively; their respective red false-color images showing the densified CNT carpets are presented on the right side (Fig. 4c and e). Areal coverage of CNTs grown on Pristine-20 and Damaged-20 using ImageJ^44^ for analysis are 18.05% and 27.10%, respectively. The results show that density of CNTs grown from catalysts on Damaged-20 is higher than CNTs obtained on Pristine-20. These results are consistent with data presented in Fig. 3, whereby coverage of CNTs and amount of carbon deposited on SS can be evaluated. In agreement with SEM data, we observed from the pictures that for the same thickness of Al_x_O_y_ barrier layer, ion beam-damaged substrate showed higher carbon coverage on the surface than the pristine substrate. In fact, quantification of the number of CNTs on Pristine-5 and Damaged-5 using their respective SEM images revealed an average density of 5 CNTs/μm^2^ and 109 CNTs/ μm^2^, respectively. It is clear from the results that ion beam bombardment improves CNT density. Also, TEM images of CNTs obtained from catalysts supported on Pristine-20 and Damaged-20 (Fig. 5) confirm that the structures are primarily CNTs and not nanofibers due to absence of a stacked cone or bamboo-like morphology along the inner cavity of the tube. Representative high-magnification TEM images of the wall structure of CNTs presented as inserts in Fig. 5a reveal that ion beam bombardment of SS prior to Al_x_O_y_ and Fe depositions yield CNTs with higher structural quality. The insert in Fig. 5a shows the presence of substantial defects on the wall of a CNT grown on Pristine-20 whereas a wall structure that has less defects is observed for the CNT grown on Damaged-20 as shown in Fig. 5b. It is apparent from the results that the nature of the underlying layer still affects the growth properties of CNTs even for catalyst substrates with relatively thicker barrier layer.

### Effects of ion beam bombardment

**Figure 6:**
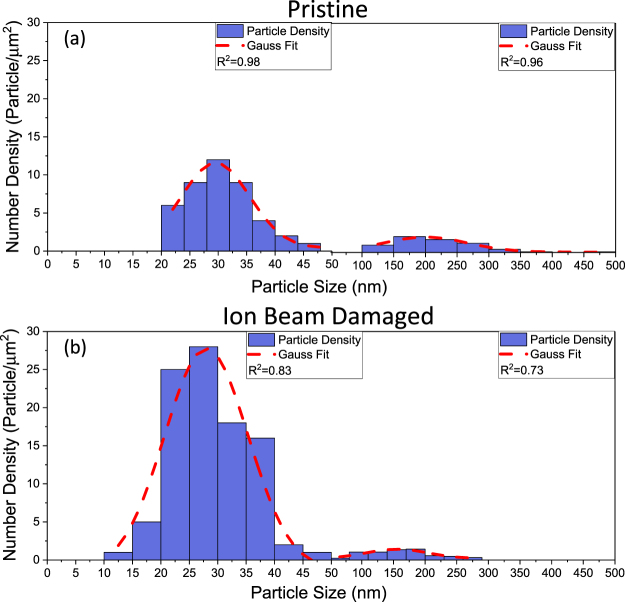
Catalyst evolution on Pristine-0 and Damaged-0. Particle size distributions (PSDs) of catalyst particles formed after CVD growth on Pristine-0 (**a**) and Damaged-0 (**b**).

To isolate the role of ion beam bombardment in CNT growth, we investigated the evolution of catalysts on pristine and ion beam-damaged substrates without an alumina layer. Figure 6 shows histograms of particle size distributions (PSDs) formed on Pristine-0 and Damaged-0 after the CCVD process. From histograms obtained from analysis of SEM images of SS surfaces using ImageJ^44^, it is apparent that PSDs of catalyst supported on Prisine-0 and Damaged-0 follow a bimodal distribution. The first modal peak of particles on Pristine-0 and Damaged-0 is centered ~30.58 nm and ~28.80 nm, respectively. Particles in the first mode are believed to be formed during the annealing step (H_2_, 750 °C, 10 min) and may have experienced ripening during the growth step (C_2_H_4_, H_2,_ Ar, 750 °C, 10 min). The position of the second modal peak of Pristine-0 and Damaged-0 indicates the presence of large particles or features with average diameters (or lateral distances) of ~216.76 nm and ~163.00 nm, respectively. Particles in the second mode appear to be mostly composed of Fe film still wetted to the substrate surface, and are considered unsuitable for CNT growth.

Although the number density of catalyst particles on Pristine-0 and Damaged-0 for the second mode is somewhat the same, there is a stark difference in the particle number density formed on Pristine-0 and Damaged-0 for the first mode. Number density of catalysts on Pristine-0 is 43 particle/µm^2^ while that on Damaged-0 is 96 particle/µm^2^. Some of the catalyst nanoparticles imaged in Fig. 2a (pristine SS) are wetted to the surface and not spherical in shape, indicating the Fe film was not fully de-wetted during the annealing process, while Fig. 2e (damaged SS) shows particles that are spherical in shape. Like alumina, ion beam bombardment plays an important role in enhancing particle formation in the size range suitable for CNT growth during annealing of the deposited Fe film. However, presence of a thin Al_x_O_y_ barrier layer is still required for CNT growth. Ion beam damage of SS increases the particle number density of particles less than 50 nm by a factor of 2. The observed decrease in average particle size of particles formed on damaged SS demonstrates that ion beam bombardment plays a significant role in not only catalyst dewetting, but also catalyst stability. Based on our previous study,^30^ we attribute the improved properties of ion beam-damaged SS to the increased porosity on the surface of SS prior to Al_x_O_y_ deposition. We conclude from our results that ion beam bombardment of SS favors formation and stability of catalyst particles during CCVD, which contributes to CNT growth efficiency.

### Catalyst evolution on pristine and ion beam-damaged SS with an Al_x_O_y_ barrier layer

**Figure 7:**
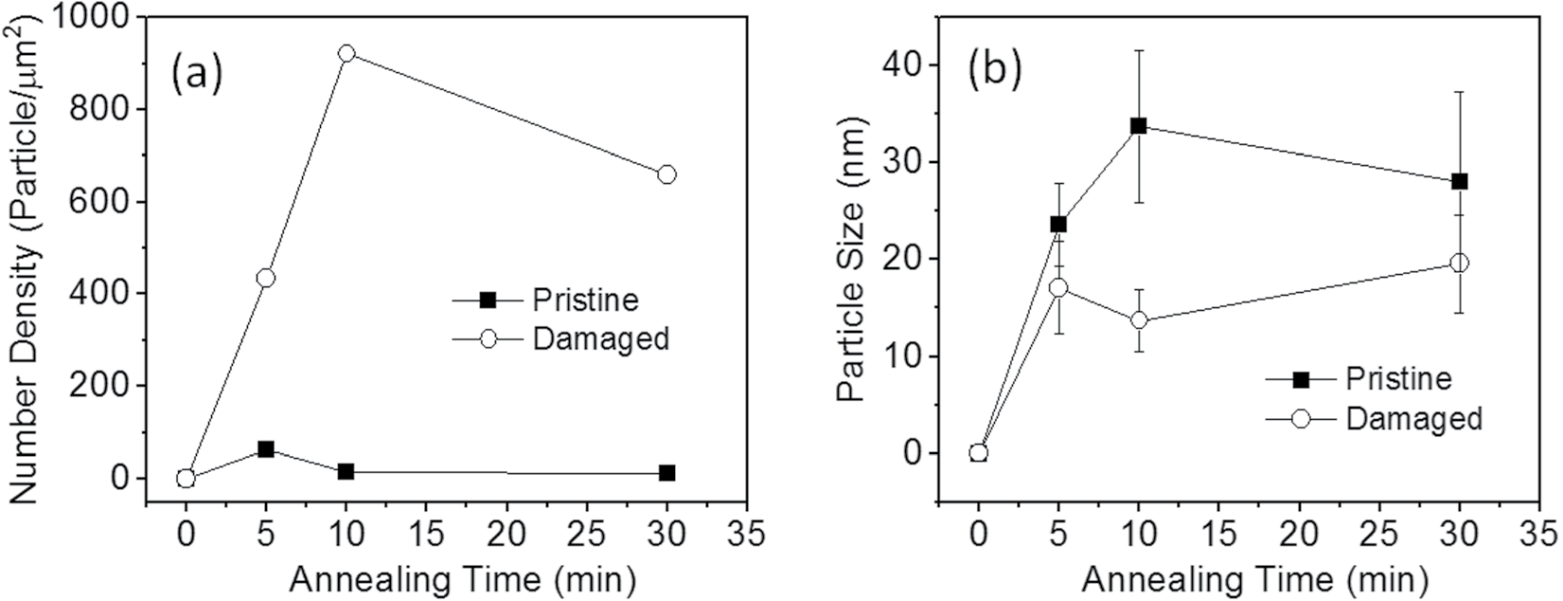
Catalyst evolution on Pristine-5 and Damaged-5 during annealing. (**a**) Plots of number density of particles as a function of annealing time. (**b**) Plots of catalyst particle size as a function of annealing time. The data were adapted from SEM images presented in supporting information.

The combined effect of ion beam bombardment of SS and an Al_x_O_y_ barrier layer on the evolution of deposited Fe catalyst film was studied. To isolate the Ostwald ripening event, an annealing study was carried out on Pristine-5- and Damaged-5-supported catalysts in the absence of the feedstock (250 sccm Ar/250 sccm H_2_). Figure 7 shows plots of number density of particles and average catalyst size as functions of annealing time. The data were derived from SEM data presented in the Supporting Information. Number density of particles on Damaged-5 after annealing for the different times is significantly higher than particles on Pristine-5 (Fig. 7a). In fact, after annealing for 5, 10, and 30 min, number density of particles on Pristine-5 was 62, 16, and 13 particle/μm^2^, respectively. In contrast, number density of particles on Damaged-5 after annealing for 5, 10, and 30 min was 435, 922, and 659, respectively. Note that while number density of particles on Pristine-5 decreases with time, catalysts supported on Damaged-5 exhibit dramatic increases in number density, with an increase of more than 50% between 5 and 10 min. In addition, we observed that catalyst particles on Damaged-5 have a lower average size in comparison to Pristine-5 (Fig. 7b). After annealing for 5, 10, and 30 min, average size of particles on Pristine-5 was 23.6, 33.8, and 28.1 nm, while the average size of particles on Damaged-5 was 17.1, 13.7, and 19.6 nm, respectively. These results show that ion beam bombardment of SS in combination with an Al_x_O_y_ barrier layer dramatically improves particle formation (or dewetting), and has the ability to suppress Ostwald ripening or enhance catalyst stability. We, therefore, attribute the improved growth behavior of Damaged-5, Damaged-10, and Damaged-20 to the synergistic effect of ion beam bombardment and thin Al_x_O_y_ barrier layer. Creation of surface porosity and a defective surface by ion beam bombardment, and a basic surface by Al_x_O_y_ deposition, may enhance the stability of catalyst particles on the surface and limit their mobility throughout the annealing and growth processes.

## Discussion

We emphasize that since Damaged-0 does not support CNT growth (Fig. 2), this suggests that modification via ion beam bombardment alone under the conditions used in this study is incapable of transforming SS from an “inactive” to an “active” substrate. In the case of sapphire^28,30^, it was possible to achieve complete transformation after ion beam damage alone, because the process created both surface porosity and increased basicity. Creation of cationic vacancies during ion beam damage, which was evidenced by an increased O/Al ratio, accounted for increased basicity. Ion beam bombardment is not expected to change the surface energy of the basic component of SS due to its composition, thus for complete transformation of SS, an alumina layer that provides a basic environment is required in combination with ion beam bombardment. However, we emphasize that improved CNT growth efficiency observed on ion beam-damaged SS with the same Al_x_O_y_ thickness, as evidenced by improved CNT areal density, growth rate, and surface coverage of CNTs, demonstrates the critical role of ion beam bombardment in the transformation of “inactive” substrates to “active” substrates.

To rationalize the observed synergistic effect of thin Al_x_O_y_ barrier layer and ion beam bombardment, we took a closer look at the effect of each factor on CNT catalysis. Fe catalyst has a strong interaction with surface oxygen atoms of Al_x_O_y_ and forms Fe^2+^ and Fe^3+^ interface states on Al_x_O_y_, which is believed to enhance catalyst stability and inhibit severe sintering of the catalyst.^45^ In addition, our previous study revealed a complex interdependence between Ostwald ripening rates, subsurface diffusion rates, and porosity; and that catalytic activity of Fe is maximized on Al_x_O_y_ support because of the high porosity of Al_x_O_y_, mild subsurface diffusion of Fe, and presence of surface hydroxyl groups^29,32,46,47^. It is apparent from these studies and our results that Al_x_O_y_-Fe interactions favor increased particle formation and CNT nucleation density as well as CNT growth over a broad range of CCVD conditions. In the case of ion beam bombardment of substrates, prior studies indicate the process induces atomic vacancies and interstitials thereby introducing porosity and surface roughness^28,34,35^. These changes in the substrate caused by ion beam bombardment has been reported to improve adhesion between ion beam-damaged substrates and deposited films^34^. We, therefore, explain the observed CNT growth enhancement after SS modification as follows: The increased surface roughness and porosity caused by ion beam bombardment enhances intermixing between SS and alumina film, resulting in improved coating of the thin barrier layer. Consequently, a higher fraction of Fe catalyst interacts directly with Al_x_O_y_ in Damaged-5, Damaged-10, and Damaged-20, and benefits from the positive role of Al_x_O_y_ discussed above in comparison to their pristine counterparts. As shown in Fig. 6, even particles that are in direct contact with ion beam damaged surface in the absence of alumina exhibit higher stability that pristine substrates due to the presence of a porous upper layer. The observed reduction in the thickness of the barrier layer required for CNT growth may be due to the contribution of the upper porous layer created by ion beam damage, which creates a thicker alumina-like layer (in terms of surface diffusion properties) required for favorable catalyst-support interactions.

## Conclusions

In summary, we have demonstrated the impact of surface modification (changes in surface structure and Lewis basicity) of 316 SS, a known inactive metallic substrate for CNT growth, on CNT growth behavior from deposited Fe catalyst. Surface modification of SS via ion beam bombardment can in fact decrease the barrier-layer thickness required for unaligned CNT growth to ~5 nm and dense CNT carpet growth to 20 nm. An Fe catalyst supported on damaged SS with an Al_x_O_y_ barrier layer shows improved activity, and the resulting CNT arrays have higher quality and density in comparison to a pristine substrate (without ion beam damage) with an Al_x_O_y_ barrier layer. From annealing studies, it is clear that while ion beam damage alone improves particle formation (or dewetting) and catalyst stability, the combined effect of ion beam bombardment and an Al_x_O_y_ barrier layer is significantly higher. This new approach can potentially benefit applications that require high electron transport between CNTs and metallic substrates.

## Experimental

### Materials and Preparation

316 SS substrates used as catalyst supports were modified by ion beam bombardment in an ion beam sputter deposition and etching system (IBS/e) obtained from South Bay Technology. The substrates were placed directly opposite the Ar ion source (spot size ~3 mm) with adjustments made to ensure the beam line is perpendicular to the substrate. Ion beam damage was conducted for 10 min at an acceleration voltage of 6 kV and a beam current of 3.5 mA. The total ion dose was calculated to be 1.85 × 10^18^ cm^−2 ^^28^. Thereafter, damaged and pristine SS substrates were deposited with different thicknesses of Al_x_O_y_ films (0, 5, 10, and 20 nm). A thin Fe catalyst film with a nominal thickness of 1 nm was then deposited on each SS-supported Al_x_O_y_ layer. The Al_x_O_y_ and Fe films were sequentially deposited on substrates in the IBS/e without exposure to air.

### CVD Growth

CNT growth was carried out at atmospheric pressure using an EasyTube 101 CVD system (CVD Equipment Corporation) equipped with a LabView-based process control software, a static mixer for optimum gas mixing, and a precise temperature control system. A typical growth run involved heating the catalyst sample to 750 °C at a rate of 50 °C/min in a flowing Ar atmosphere. At the growth temperature, the sample was exposed to a copious amount of H_2_ in combination with Ar for 10 min to reduce the catalyst; respective flow rates were 250 standard cubic centimeters per minute (sccm) H_2_ and 250 sccm Ar. Thereafter, CNT growth was initiated by introducing the growth gas mixture (100 sccm C_2_H_4_, 250 sccm H_2,_ and 250 sccm Ar). At the end of growth, the samples were rapidly cooled in H_2_, followed by slow cooling to room temperature in 700 sccm Ar.

### Characterization

Raman spectra of products were collected at multiple spots from the samples using a Renishaw inVia Raman microscope with a 532-nm laser as the excitation source. Growth products and catalyst morphology were characterized with a Hitachi S5200 field-emission scanning electron microscope (SEM) operated at 5 kV. Transmission electron microscopic (TEM) images were obtained using FEI Tecnai F20 XT operating at 200 kV. The samples were dispersed in ethanol by sonication for 5 min, dropped on copper microgrid coated with lacy carbon film.

## Electronic supplementary material

Figure SI 1
