# Supplementary material for: Rational Modification of a Metallic Substrate for CVD Growth of Carbon Nanotubes
Source: Sci Rep. 2018 Mar 12;8:4349. doi: 10.1038/s41598-018-22467-7 (PMC5847581; doi:10.1038/s41598-018-22467-7)
Supplement: Supplementary file 1 — Figure SI 1 [file 41598_2018_22467_MOESM1_ESM.docx]

SUPPLEMENTARY INFORMATION

**Rational Modification of a Metallic Substrate for CVD Growth of Carbon Nanotubes**

Xu Li, Montgomery Baker-Fales, Haider Almkhelfe, Nolan R. Gaede, Tyler S. Harris, and Placidus B. Amama^^[[1]](#footnote-1)^^

*Department of Chemical Engineering, Kansas State University, Manhattan, KS 66506*

**Figure SI 1.** Catalyst evolution on Pristine-5 and Damaged-5 during annealing. Upper panels: SEM images of catalyst particles on Pristine-5 (with inserts of PSDs) after annealing for 5 min (a), 10 min (b), and 30 min (c). Bottom panels: SEM images of catalyst particles on Damaged-5 (with inserts of PSD) after annealing for 5 min (d), 10 min (e), and 30 min (f).

1. [↑](#footnote-ref-1)
